# Draft Genome Sequence of Extended-Spectrum β-Lactamase-Producing *Klebsiella pneumoniae* Isolated from a Patient in Lebanon

**DOI:** 10.1128/genomeA.00121-14

**Published:** 2014-02-20

**Authors:** Sima Tokajian, Jonathan A. Eisen, Guillaume Jospin, Anna Farra, David A. Coil

**Affiliations:** a Lebanese American University, Department of Natural Sciences, School of Arts and Sciences, Byblos, Lebanon; b University of California Davis Genome Center, Davis, California, USA; c Lebanese American University, School of Medicine, Byblos, Lebanon

## Abstract

We present the draft genome sequence of extended-spectrum β-lactamase (ESBL)-producing *Klebsiella pneumoniae* isolated from a stool sample collected from a patient admitted for a gastrointestinal procedure. The draft genome sequence consists of 86 contigs, including a combined 5,632,663 bases with 57% G+C content.

## GENOME ANNOUNCEMENT

The emergence of extended-spectrum β-lactamase (ESBL)-producing bacteria is now a critical concern. β-lactamases are plasmid-mediated enzymes which are able to hydrolyze oxyimino-cephalosporins and aztreonam and constitute an increasingly important mechanism of antimicrobial resistance among nosocomial Gram-negative pathogens (1).

*Klebsiella pneumoniae* is among the most important causes of both hospital- and community-acquired serious bacterial infections in humans. ESBL-producing organisms remain important causes of the failure of therapy with cephalosporins and have serious infection control consequences. ESBL-producing *K. pneumoniae* is most often involved in urinary and respiratory tract infections (2). The ESBL genes are mostly carried by plasmids (3), and most ESBLs can be divided into three genotypes: TEM, SHV, and CTX-M (4). In this study, we sequenced one ESBL-producing *K. pneumoniae* strain, LAU-KP1, isolated from a stool sample from a patient admitted for a gastrointestinal procedure/surgery at the University Medical Center-Rizk Hospital (UMCRH) in Lebanon.

Illumina paired-end libraries were made from sonicated DNA using a TruSeq DNA sample prep version 2 kit (Illumina). Fragments between 300 and 600 bp were selected using a Pippin Prep DNA size selection system (Sage Science). A total of 4,834,956 paired-end reads were generated on an Illumina MiSeq, at a read length of 250 bp. Quality trimming and error correction of the reads resulted in 4,220,969 high-quality reads. All sequence processing and assembly were performed using the a5 assembly pipeline. This pipeline automates the processes of data cleaning, error correction, contig assembly, scaffolding, and quality control (5). The initial assembly produced 86 contigs, for which no scaffolding was obtained. The final collection of contigs was submitted to GenBank. The final draft genome sequence consists of a combined 5,632,663 bases with 57% G+C content. Automated annotation was performed using the RAST annotation server (6). *K. pneumoniae* LAU-KP1 contains 5,424 predicted coding sequences and 117 predicted RNAs.

### Nucleotide sequence accession numbers.

This whole-genome shotgun project has been deposited at DDBJ/EMBL/GenBank under the accession no. AYQE00000000. The version described in this paper is the first version, accession no. AYQE01000000.
